# Analysis of the Beneficial Effects of Probiotics on the Gut–Prostate Axis Using Prostatic Co-Culture Model

**DOI:** 10.3390/foods13223647

**Published:** 2024-11-16

**Authors:** Sara Ferrari, Rebecca Galla, Simone Mulè, Francesca Uberti

**Affiliations:** 1 Laboratory of Physiology, Department for Sustainable Development and Ecological Transition, 13100 Vercelli, Italy; 2 Noivita Srls, Spin Off, University of Piemonte Orientale, Via Solaroli 17, 28100 Novara, Italy

**Keywords:** gut–prostate axis, probiotics, oral supplementation, co-culture prostatic model

## Abstract

The link between the gut environment and the prostate has recently been proposed as a potential therapeutic approach for treating benign prostatic hyperplasia (BPH). Therefore, this study examined the advantages of a novel oral probiotic supplement to improve intestinal health and treat BPH. A 3D intestinal barrier model that simulated oral intake was used to analyse the combined regulative abilities of *Bifidobacterium longum* and *Bifidobacterium psychaerophilum*. Then, a co-culture prostatic model was used to investigate the biological consequences of the combination under conditions mimicking BPH. The results show the connection between the gut microbiome and prostate disease since the probiotics successfully modulate the primary mechanism involved in the pathogenesis of BPH. Indeed, after the intestinal passage, the mediators released from *B. longum* and *B. psychaerophilum* induced a substantial decrease in reactive oxidative species of about 6 times and inflammation (about 5 times regarding interleukine-6 and 10) and a sharp increase in testosterone and serotonin levels (about 95%). Further, proliferation and BPH principal mediators (such as androgen and dihydrotestosterone) were highly affected and nearly restored to physiological levels. Thus, BPH can be directly affected by probiotic supplementation; specifically, *B. longum* and *B. psychaerophilum*, in combination, seem able to promote the mitigation of this disease.

## 1. Introduction

The prostate gland is a nut-shaped organ on the bladder’s neck before the rectum, where the male urethra crosses it. The function of the prostate is to generate sperm. In addition to that, the muscles connected to the prostate gland aid in ensuring that the semen is forced up against the urethral walls and subsequently released during ejaculation [1]. The periphery, transition, and core zones are the three distinct histological zones that comprise the human prostate gland. About 70% of the prostate’s tissue is found in the peripheral zone, while just 5% is found in the transition zone. The transitional zone is mainly prevalent in elderly men and is relatively more inconspicuous in younger men, although it appears to be generally enlarged due to benign prostatic hyperplasia (BPH) [2]. Indeed, the non-malignant abnormal growth of the prostate is known as BPH and leads to a restriction of the urethra and can induce incapacitating symptoms related to urination. This illness is more common in elderly males; in 2000, there were 51.1 million instances worldwide; in 2019, there were over 94 million cases [3]. Clinically, prostate enlargement results from the stimulation of cells in the transitional zone around the urethra by testosterone and its metabolites [4]. Many factors, such as increased sympathetic nerve activity, hormonal alterations, metabolic syndrome, and tissue remodelling brought on by ageing, are likely involved in the development and progression of BPH, despite its high prevalence and socioeconomic significance [5]. The prostate’s growth, maintenance, and secretory function are stimulated explicitly by the continuous presence of several hormones and growth factors, the most important of which is serum testosterone. Indeed, changes in androgen levels are thought to be the main factor behind the onset and advancement of prostate disorders, such as BPH. Prostate cell proliferation is androgen dependent. Thus, the cornerstone of pharmaceutical treatment for BPH is androgen signaling inhibition [6]. In this setting, pharmaceutical medications, including α-adrenergic blockers and 5α-reductase inhibitors, have shown promise in the treatment of BPH; nevertheless, these treatments are not without adverse effects and can cause insomnia, erectile dysfunction and even prostate cancer [7]. However, current evidence suggests that higher levels of testosterone are linked to the pathogenic process through the stimulation of inflammation within the tissue. Still, they are not involved in BPH development [8]. It has been shown that having a prolonged prostatic inflammatory state in and of itself raises the likelihood of BPH developing more quickly [9].

As an outstanding achievement, one of the main causes of systemic inflammation that leads to BPH is alteration in the intestinal microbiome [10]. The relationship between gut microbiota and urologic disease has been the subject of numerous investigations, emphasising the aetiology and treatment of these prevalent prostatic dysfunctions [10,11]. The concept of a “gut–prostate axis” is supported by the significant indirect internal connections between the gut microbiome and prostatic illness, such as the modulation of prostate enlargement by short-chain fatty acids (SCFAs) derived from gut bacteria [12]. Additionally, research has indicated that gut barrier permeability and prostate illness may be primarily linked to proinflammatory circumstances. Lipopolysaccharides (LPSs) and other proinflammatory cytokines can leak into the systemic circulation when the intestinal barrier is disrupted. LPSs stimulate proinflammatory cytokines, which can trigger the BPH/inflammatory process and induce inflammation and tissue remodeling [13,14]. The term “gut–prostate axis” was initially used in 2005 to refer to the intimate connection between the prostate and the gut during treating prostatitis. Since then, several studies have demonstrated a link between prostate illness and quantitative and functional abnormalities of the gut microbiota and/or intestinal barrier [15]. Clinical investigations on this subject are becoming more and more prevalent, and they indicate that inflammatory and functional bowel disorders are predisposing risk factors for the development of prostate pathology.

Therefore, this physiological phenomenon is a possible target for diagnosis and treatment since gut microbiota may be crucial to the pathophysiology of BPH and clinical development [10,16]. Prostate inflammation caused by specific bacteria can be persistent and increase the production of proinflammatory cytokines. These findings imply that a significant contributing factor to the pathogenesis of BPH may be ecological dysbiosis of the microbiota in the prostatic fluid [17]. Acute and persistent inflammation may be a pathophysiological mechanism by which pathogenic bacteria contribute to BPH [18]. The relationship between stool SCFA levels in elderly men with BPH and healthy controls was examined, showing that compared to the control group, patients with BPH had considerably more significant amounts of branched SCFAs, such as isobutyric acid and isovaleric acid [19]. Clinical studies have demonstrated probiotics’ efficacy in treating bacterial prostatitis, as one trial utilised probiotic supplements alongside antibiotics for chronic bacterial prostatitis management [20]. Given the involvement of inflammation in the pathophysiology of BPH and the possible role of probiotics in preventing or alleviating gut or other systemic diseases, it appears that probiotics are crucial for maintaining intestinal barrier function [21]. Research published in the literature indicates that treating prostatitis with antibiotics alone is insufficient to cure it. Therefore, by decreasing the likelihood of recurring bacterial and viral illnesses, probiotic supplementation can help repair the loss of beneficial bacteria in the gut microbiota [20]. Furthermore, using a probiotic bacterial strain orally to change the gut microbiome has been demonstrated to impact the prostate’s inflammatory environment [22]. Investigating the efficacy of various probiotic strains in reducing the progression of BPH appears promising. For example, *Bifidobacteria* members are anaerobic, with Gram-positive bacilli colonising the human digestive system and the mouth cavity. Their presence and abundance are a marker for health. Probiotics are known to provide a wide range of health advantages. SCFAs and lactate, specially produced by Bifidobacteria, are believed to prevent disease by boosting immunity and acidifying the intestinal environment [23].

In addition, *Bifidobacterium* species enhance intestinal flora by lowering dangerous bacteria, reducing inflammation, and preventing elevated cholesterol [24]. Furthermore, because of their generally recognised safe status and related health advantages, Bifidobacteria have been used commercially as probiotic agents [25]. Investigating the potential of different probiotic strains to halt the progression of BPH seems like a promising strategy in this context. As a result, two probiotic strains—*Bifidobacterium longum* and *Bifidobacterium psychraerophilum*—have been examined in this study.

Thus, this study intends to confirm this hypothesis by examining the primary mechanisms underlying BPH to postulate a novel human formulation, applying many probiotics to improve the ultimate target organ, and employing a gut in vitro model that mimics the gut–prostate axis. Notably, the primary effects on the prostate were examined using the co-culture prostatic model.

## 2. Materials and Methods

### 2.1. Agents Preparation

*Bifidobacterium longum* subsp. *longum* novaBLG1 (DSM 34338, called *B. longum* BLG1) and *Bifidobacterium psychraerophilum* Q5 (DSM 33131, called *B. psychraerophilum* Q5) donated by Probionova (Lugano, Switzerland) were prepared just before usage. Specifically, a new pack of the probiotics was mixed and reconstituted into Dulbecco’s Modified Eagle’s Medium (DMEM) without red phenol (Merck Life Science, Rome, Italy), supplemented with 0% Fetal Bovine Serum (FBS, Merck Life Science, Rome, Italy), 50 IU/mL penicillin-streptomycin (Merck Life Science, Rome, Italy) and 2 mM L-glutamine solution (Merck Life Science, Rome, Italy) before each stimulation. The samples were diluted in culture medium before each test, which was conducted in triplicate, until the final concentration of 1 × 10^9^ colony-forming units (CFU)/mL probiotic of 1 × 10^9^ colony-forming unit (CFU)/mL probiotics and 1 × 10^9^ CFU/mL probiotics for *B. psychaerophilum Q5* was achieved. To induce the prostatic hyperplasia condition, the cells and the prostate model were exposed to Dihydrotestosterone (DHT, Merck Life Sciences, Rome, Italy) for 4 days. The substance was diluted with DMEM medium devoid of red phenol and reconstituted in DMSO (Merck Life Sciences, Rome, Italy). Then, 0% FBS, 50 IU/mL penicillin-streptomycin (Merck Life Science, Rome, Italy), and 2 mM L-glutamine solution (Merck Life Science, Rome, Italy) were added to the medium to reach a final concentration of 10 nM.

### 2.2. Cell Culture

A standard intestinal epithelial barrier model is the human colorectal cancer cell line CaCo-2 (ATCC, Manassas, VA, USA) [26]. This cell line was maintained in an incubator set at 37 °C and 5% CO_2_ and cultured in Dulbecco’s Modified Eagle’s Medium/Nutrient F-12 Ham (DMEM-F12, Merck Life Science, Rome, Italy) containing 10% FBS (Merck Life Science, Rome, Italy), 2 mM L-glutamine and 1% penicillin-streptomycin (Merck Life Science, Rome, Italy). Cells used in the studies had passage numbers between 26 and 32 to keep the integrative nature of paracellular permeability and transport properties similar to how people’s intestines absorb food after eating it [21]. The cells were plated 1 × 10^4^ cells in 96-well plates to study cell viability; 2 × 10^4^ cells were plated on 6.5 mm Transwell^®^ (Corning^®^ Costar^®^, Merck Life Science, Rome, Italy) in a 24-well plate to perform the integrity analyses and SCFA and butyric acid determination. Further, 2 × 10^4^ cells were plated on 6.5 mm Transwell^®^ (Corning^®^ Costar^®^, Merck Life Science, Rome, Italy) in a 24-well plate to recreate the gut–prostate axis; specifically, Caco-2 cells were plated on the membrane, while RWPE-1 and WPMY-1 cells were plated on the bottom of the well to create a co-culture prostatic model. The cells were synchronised eight hours before the stimulation by being incubated at 37 °C with DMEM devoid of red phenol and supplemented with 0.5% FBS (Merck Life Science, Rome, Italy), 2 mM L-glutamine, and 1% penicillin-streptomycin (all from Merck Life Science, Rome, Italy).

According to a published procedure, two cell lines were employed to create a co-culture prostatic model [27]. RWPE-1 and WPMY-1 were obtained from ATCC (Manassas, VA, USA). RWPE-1 was cultured in Keratinocyte serum-free medium (K-SFM, Thermofisher, Waltham, MA, USA) containing 0.05 mg/mL of bovine pituitary extract (BPE, Merck Life Science, Rome, Italy) and 5 ng/mL of epidermal growth factor (EGF, Merck Life Science, Rome, Italy) and maintained in an incubator at 37 °C and 5% CO_2_. WPMY-1 was cultured in DMEM (Merck Life Science, Rome, Italy) supplemented with 5% FBS (Merck Life Science, Rome, Italy), 1 mM of sodium pyruvate, 4 mM L-glutamine solution (Merck Life Science, Rome, Italy) and 1% penicillin-streptomycin (Merck Life Science, Rome, Italy) and maintained in an incubator at 37 °C and 5% CO_2_. For the co-culture, a 1:1 mixture of K-SFM and DMEM was supplemented with 2.5% FBS, 0.025 mg/mL BPE and 2.5 ng/mL EGF, 0.5 mM sodium pyruvate, 2 mM l-glutamine, and 0.5% penicillin-streptomycin. Specifically, the cells were plated on the bottom of 24-well plate containing the Transwell^®^ system with Caco-2 cells.

### 2.3. Experimental Protocol

The experiments were divided into two phases to assess the possible positive benefits of probiotic strains in reducing BPH following oral ingestion: the first examined probiotics’ capacity to affect intestinal barrier activities without causing negative side effects, while the second examined the direct effects of probiotic metabolites on a co-culture prostatic model that replicated BPH scenarios. The CaCo-2 cell line was utilised to exclude the cytotoxicity of *B. longum* BLG1 and *B. psychaerophilum* Q5, alone and combined, by testing the mitochondrial metabolism using 3-(4,5-Dimethylthiazol-2-yl)-2,5-diphenyltetrazolium bromide (MTT) [28]. The MTT test was then used to confirm cell viability using the optimal concentrations of *B. longum BLG1* and *B. psychaerophilum Q5* on a 3D intestinal in vitro barrier model. Transepithelial electrical resistance (TEER) analysis was used to test individual and combined probiotic strains to ensure intestinal stability and properly maintain epithelial integrity and paracellular pathway ionic conductance in the epithelial monolayer; moreover, further integrity analyses were performed analysing the activity of the main tight junctions such Claudin-1, Occludin and ZO-1. Additionally, an ELISA test was employed to measure SCFA and butyric acid levels to confirm the function of SCFA in regulating cell signalling across the whole body. In all of these tests, the cells were treated at different times, from 2 to 6 h [29]. In addition, probiotic aggregation was evaluated on Caco-2 cells. In the second step, a gut–prostate axis was recreated. Specifically, Caco-2 cells were plated on the apical and the co-culture prostatic at the basal compartments of the Transwell^®^ system, respectively. The significance of this passage was that it enabled the further investigation of the metabolites accountable for improving the BPH condition by analysing the volatile profile of a biological system, which was achieved by evaluating volatile organic compounds such as butyric acid. The effects of the probiotic strains on the hyperplasic co-culture prostatic model were therefore examined after the co-culture prostatic model was pre-treated for four days with 10 nM DHT (Merck Life Science, Rome, Italy) to simulate BPH in vitro [27]. The stimulation with *B. longum* BLG1 and *B. psychaerophilum* Q5 lasted 24 h, the right amount of time to mimic the right treatment dose. After the stimulation, the production of reactive oxygen species (ROS) and the activation of antioxidant molecules such as superoxide dismutase (SOD) were studied, along with the reduction in inflammatory processes. The main cytokines involved in BPH, such as Tumor Necrosis Factor-alpha (TNF-α), interleukin (IL)-6, and IL-10, were studied. The molecular pathways involved in BPH were also investigated. Increased DHT production up-regulates the androgen receptor (AR), while decreased serotonin production and 5-hydroxytryptamine receptor 1a (5-Htr1a) levels are caused by out-of-control prostate cell growth. These changes led to more prostate-specific antigens (PSAs), so this measure was also scrutinised. In addition, the reduction in proliferative activity in human hyperplasia was studied using crystal violet staining and the quantification of Ki67, a marker of growth cells.

### 2.4. Cell Viability

According to standard procedures [26], the MTT-based in In Vitro Toxicology test Kit (Merck Life Science, Rome, Italy) test was used to check cell viability and rule out the cytotoxic effects of probiotics. After stimulations, cell viability was found by using a spectrometer (Infinite 200 Pro MPlex, Tecan, Männedorf, Switzerland) to measure absorbance at 570 nm with correction at 690 nm. The results were then compared to cells that had not been treated (baseline 0%), and the results were reported as means ± SD of five separate experiments that were conducted three times.

### 2.5. In Vitro Intestinal Barrier

Using a 24-well plate and 0.4 μm pore polycarbonate membrane inserts (Corning^®^ Costar^®^, Merck Life Science, Rome, Italy), 2 × 10^4^ Caco-2 cells were plated on 6.5 mm Transwell^®^ to construct an intestinal barrier in vitro model. For 21 days before the simulations, cells plated on the Transwell^®^ insert were cultured in a complete medium that was replaced every other day on both the apical and basolateral sides [30]. The medium had been adjusted to pH 6.5 on the apical side before stimulation, the same as the pH in the small intestinal lumen. However, a pH of 7.4 on the basolateral side indicated blood [30]. This in vitro model is extensively utilised and endorsed by the European Medicines Agency (EMA) and Food and Drug Administration (FDA) to forecast the absorption, metabolism, and bioavailability of numerous substances following oral administration by humans [31,32]. The intestinal barrier’s integrity was verified following probiotic delivery by utilising EVOM3 (World Precision Instruments, Sarasota, FL, USA) to measure the TEER. This experiment was conducted every two days for 21 days to evaluate the appropriate epithelial functions until the TEER value was ≥400 Ωcm^2^ before the stimulation [33]. On day 21, the apical and basolateral mediums were altered to create varied pH conditions, as previously mentioned [34]. Probiotics may be susceptible to antibiotics, so the cells were rinsed with Hanks’s Balanced Salt solution (HBSS) and remained in an antibiotic-free medium throughout the simulation [35]. Before the experiment began, the TEER values were tested again to confirm that the readings had stabilised after the cells had been maintained at 37 °C and 5% CO2 for 15 min. Before the following analysis, which included tests to measure cell viability and the evaluation of total SCFA production and butyric acid production—a particular metabolite with second messenger properties that can maintain the host’s everyday health and prevent many diseases—the cells were stimulated with probiotic strains for two to six hours, which is the time frame during which the intestine performs its physiological functions [36]. Finally, the adhesion capacity of the probiotics was also evaluated to confirm that the probiotics do not cross the intestinal barrier but rather adhere to it.

### 2.6. Human Short Chain Fatty Acid (SCFA) ELISA Kit

Following the manufacturer’s instructions, the Human ScFA (short-chain fatty acids) Elisa Kit (FineTest, Palm Coast, FL, USA) measured basolateral medium SCFA levels. After cell lysis with PBS1X and protease inhibitor, 50 µL of material was added to the plate and incubated at 37 °C for 45 min. After washing the sample wells twice, 100 µL of SABC working solution was added to the plate. Following a 30 min incubation at 37 °C, the plate was rewashed, and 90 µL of 90 µL TMB substrate solution was added for 10–20 min at 37 °C. By comparing the results to a standard curve (ranging from 0 to 2000 pg/mL), the absorbance was measured at 450 nm using a spectrophotometer (Infinite 200 Pro MPlex, Tecan, Männedorf, Switzerland). The concentration was given in pg/mL. The study reported means ± SD (%) compared to control (0 line) from five triplicate tests.

### 2.7. Butyric Acid Quantification

Following the manufacturer’s instructions, the ELISA kit (Cloud-Clone, Wuhan, China) was used to analyse the butyric acid generated on the basolateral medium [37]. An Infinite 200 Pro MPlex plate reader (Tecan, Männedorf, Switzerland) assessed each sample’s absorbance at 450 nm after adding the stop solution. Outcomes (ODs) were interpolated using a standard curve (0–10,000 pg/mL) and presented as averages ± SD (%) versus the control of five triplicate experiments.

### 2.8. Surface Hydrophobicity and Aggregation Activity

Surface hydrophobicity and aggregation assay were conducted using the methodology outlined in the literature [38]. PBS was used as the control, while 0.4 mL of xylene was mixed with 4 mL of suspension with probiotics and then placed for 15 min to obtain the aqueous phase. The absorption values of the sample and control were taken at the wavelength of 600 nm. Each analysis was performed in triplicate with 10 parallel samples. The following formula calculated surface hydrophobicity:

$$Surface\;hydrophobicity=\frac{{OD}_{600}\left(control\right)-{OD}_{600}(test)}{{OD}_{600}\left(control\right)}\times 100\%$$

OD_600_ (control): the absorption value of the control.
OD_600_ (test): the absorption value of the sample.

Next, 0.1 mL of probiotic suspension was mixed with 2.9 mL of PBS, and then absorption was detected at 600 nm. The following equation defines aggregation activity:

$$Aggregation\;activity=\frac{{OD}_{600}\left(0\right)-{OD}_{600}(t)}{{OD}_{600}\left(t\right)}\times 100\%$$

OD_600_ (0): the absorption value of the probiotics after blending with the buffer.
OD_600_ (t): the absorption value of the probiotics blending with the buffer after 2 h.

### 2.9. Tight Junction Analysis

Following the manufacturer’s instructions, the CaCo-2 lysates were tested for occludin activity analysis using the Human Occludin (OCLN) ELISA Kit (MyBiosource, San Diego, CA, USA), Claudin-1 levels analysis using the Cusabio Technology LCC ELISA Kit (Huston, Houston, TX, USA), and ZO-1 activity analysis using the human tight junction protein 1 (TJP1) ELISA kit (MyBiosource, San Diego, CA, USA). A spectrophotometer (Infinite 200 Pro-MPlex, Tecan, Männedorf, Switzerland) was used to measure the absorbance at 450 nm. The data were compared to the standard curve, which ranges from 0 to 1000 pg/mL for ZO-1 and claudin-1 and from 0 to 1500 pg/mL for occludin. The results were given as a percentage (%) concerning the control (0 line) of five separate tests performed in triplicates.

### 2.10. Co-Culture Prostatic Model

The advantageous effects of a probiotic strain on BPH following oral administration were investigated by developing an in vitro co-culture prostatic model, as documented in the literature [27]. Briefly, two prostate cell lines (RWPE-1 and WPMY-1) were seeded in a 1:1 ratio for a total of 600,000 cells/well in 12-well plates and cultured with a 1:1 mixture of K-SFM and DMEM supplemented with 2.5% FBS, 0.025 mg/mL BPE and 2.5 ng/mL EGF, 0.5 mM sodium pyruvate, 2 mM l-glutamine, and 0.5% penicillin-streptomycin (all purchased from Merck Life Science, Rome, Italy). Precisely, hydrogels with 2% agarose were placed onto 12-well tissue culture plates using a 12–256 Small Spheroid template (Microtissues Inc., Providence, RI, USA). The cells were cultured for 4 days, and to mimic BPH in vitro, the co-culture prostatic model was treated with 10 nM of DHT [27]. Despite this, the cells were cleaned using Hank’s Balanced Salt Solution (HBSS) because probiotics may be susceptible to antibiotics. During the stimulation period, they were maintained in a medium devoid of antibiotics [35].

### 2.11. ROS Production

A conventional procedure based on the reduction in cytochrome C was used to quantify the superoxide anion’s release rate. A spectrometer (Infinite 200 Pro MPlex, Tecan, Männedorf, Switzerland) assessed absorbance at 550 nm in culture supernatants. O2 levels were represented as the mean ± SD (%) of nanomoles per decreased cytochrome C per microgram of protein compared to the control [39].

### 2.12. SOD ELISA Kit

SOD levels were evaluated using Cayman’s Superoxide Dismutase Assay Kit, which detects Cu/Zn, Mn, and Fe SOD [40]. SOD levels in cell lysates were evaluated against a standard curve (0.05–0.005 U/mL). Total cell lysates were collected using cold PBS1x. The samples’ absorbance was measured at 480 nm using a spectrometer (Infinite 200 Pro MPlex, Tecan, Männedorf, Switzerland). Results are presented as means ± SD (%) compared to control.

### 2.13. TNF-α ELISA Kit

The manufacturer’s instructions were to assess TNFα levels in the supernatant using a TNF-α ELISA kit (Merck Life Science, Milano, Italy) [41]. Samples were measured at 450 nm using a plate reader (Infinite 200 Pro MPlex, Tecan, Männedorf, Switzerland). Concentration was expressed as pg/mL against a standard curve (from 0 to 6000 pg/mL), and the results were expressed as a percentage (%) compared to the control (line 0).

### 2.14. IL-6 ELISA Kit

Following the manufacturer’s instructions, the IL-6 concentration in the prostatic supernatant was measured using an IL-6 ELISA kit (eBioscience, San Diego, CA, USA) [42]. The spectrometer (Infinite 200 Pro MPlex, Tecan, Männedorf, Switzerland) measured the maximum absorption wavelength at 450 nm. Concentration was expressed as pg/mL against a standard curve (0.078 to 5 pg/mL), and results were shown as a percentage (%) compared to control (line 0).

### 2.15. IL-10 ELISA Kit

According to the manufacturer’s instructions, the Human IL-10 Quantikine ELISA Kit (R&D Systems, Minneapolis, MN, USA) measured IL-10 in the co-culture prostatic model supernatants. Essentially, 100 μL was combined with IL-10 conjugates after being incubated for two hours at room temperature. At room temperature, the plate was incubated for a further two hours. To produce chemiluminescence, 100 μL of substrate solution was added after washing. Using a microplate reader, the optical density was measured at 450 nm and adjusted at 540 nm. A standard curve (from 7.8 to 500 pg/mL) was used to compute IL-10 levels, which were represented as a percentage (%) relative to the control (line 0).

### 2.16. AR ELISA Kit

Following the manufacturer’s instructions, the Human AR ELISA Kit (MyBioSource, San Diego, CA, USA) measured androgen receptor concentrations in co-culture prostatic model lysates. Lysates were produced in cold PBS1x. In summary, 100 μL was incubated at 37 °C for 1 h before adding 100 μL of Detection Reagent A for another 1 h. Detection Reagent B (100 μL) was added after washing and incubated at 37 °C for 30 min. To measure OD, 90 μL of Substrate Solution was incubated at 37 °C for 10–20 min, followed by 50 μL of Stop Solution. OD was measured at 450 nm. An AR standard curve (0.312 to 20 ng/mL) was used to compute AR levels, which were reported as a percentage (%) compared to the control (line 0).

### 2.17. Testosterone ELISA Kit

The manufacturer’s instructions were to quantify testosterone in co-culture prostatic model lysates collected by cold PBS1x using the Human Testosterone ELISA Kit (FineTest, Palm Coast, FL, USA). First, 50 μL of samples and 50 μL of biotin-labelled antibodies were incubated for 45 min at 37 °C. After washing, 100 μL of SABC Working Solution was added to each well, and the plate was incubated at 37 °C for 30 min. The plate was cleaned, and 90 μL of TMB Substrate Solution was incubated at 37 °C for 10–20 min. After washing, 90 μL of Substrate Solution was incubated at 37 °C for 10–20 min. Following the addition of 50 μL of Stop Solution, OD was measured at 450 nm to determine its colour. From a standard curve (0.313 to 20 ng/mL), testosterone was computed as a percentage (%) compared to the control (line 0).

### 2.18. 5-Alpha-Reductase ELISA Kit

Following the manufacturer’s instructions, a human 3-oxo-5-alpha-steroid 4-dehydrogenase 2 (SRD5A2) ELISA kit was used to measure 5-alpha-reductase in cold PBS1x co-culture prostatic model lysates [41]. A spectrometer (Infinite 200 Pro MPlex, Tecan, Männedorf, Switzerland) measured the maximum absorption wavelength of 450 nm, yielding concentrations in pg/mL against a standard curve (12–2400 pg/mL). The results were expressed as a percentage compared to control (line 0).

### 2.19. DHT ELISA Kit

According to the manufacturer, a Human Dihydrotestosterone ELISA Kit (Cusabio, Houston, TX, USA) was used to measure DHT in co-culture prostatic supernatants [43]. Measurements were taken at 450 nm using a spectrometer (Infinite 200 Pro MPlex, Tecan, Männedorf, Switzerland). Concentration was expressed as pg/mL against the standard curve (10–2000 pg/mL) and compared to the control (line 0).

### 2.20. PSA (Total)/KLK3 Human ELISA Kit

Based on manufacturer instructions, PSA (Total)/KLK3 Human ELISA Kit (Thermofisher, Waltham, MA, USA) measured PSA in co-culture prostatic supernatants. After adding 100 μL of the sample to each well, the plate was incubated at 4 °C for the entire night. After 1X Wash Buffer washes, 100 μL of biotinylated antibody was added to each well and incubated for 1 h at room temperature. Next, 100 μL of Streptavidin-HRP was added to each well and incubated at room temperature for 45 min. The plate was washed and 100 μL of TMB Substrate was added and incubated for 30 min in the dark at room temperature. Next, 50 μL of Stop Solution was added to a spectrometer (Infinite 200 Pro MPlex, Tecan, Männedorf, Switzerland) and read at 450 nm to measure the concentration. The results were expressed as pg/mL against the standard curve (0–2500 pg/mL) and as a percentage (%) compared to the control (line 0).

### 2.21. Serotonin ELISA Kit

Serotonin levels were measured in co-culture prostatic model lysates prepared by cold PBS1x using the ST/5-HT(5-hydroxytryptamine) ELISA Kit (FineTest, Palm Coast, FL, USA) following the manufacturer’s instructions. A 50 μL sample was treated with 50 μL of Biotin-labeled Antibody at 37 °C for 45 min. After washing, 100 μL of SABC Working Solution was added into each well and incubated at 37 °C for 30 min. After incubation, the plate was washed and 90 μL of TMB Substrate Solution was incubated at 37 °C for 10–20 min. Substrate Solution (90 μL) was incubated at 37 °C for 10–20 min after washing. Stop Solution (50 μL) was added, and OD was measured at 450 nm. From a standard curve (1.563 to 100 ng/mL), serotonin (ng/mL) was computed and expressed as a percentage (%) relative to the control (line 0).

### 2.22. 5HTR1A ELISA Kit

According to the manufacturer’s instructions, the Human HTR1A (5-hydroxytryptamine receptor 1A) ELISA Kit (FineTest, Palm Coast, FL, USA) measured 5HTR1A in co-culture prostatic model supernatants. A 50 μL sample was treated with 50 μL of Biotin-labeled Antibody at 37 °C for 45 min. The plate was incubated at 37 °C for 30 min after washing the wells and adding 100 μL of SABC Working Solution to each well. After incubation, the plate was washed and 90 μL of TMB Substrate Solution was incubated at 37 °C for 10–20 min. At 450 nm, a spectrometer reddened the OD after 50 μL of Stop Solution was added. The amount of 5HTR1A (ng/mL) was estimated from a standard curve (0.156 to 10 ng/mL) and expressed as a percentage compared to the control (line 0).

### 2.23. Crystal Violet Staining

Crystal Violet Staining was utilised following the literature [26] to assess the proliferation rate following treatment in the co-culture prostatic model. The plate (Infinite 200 Pro MPlex, Tecan, Männedorf, Switzerland) was read at 595 nm using a spectrometer. The estimated number was found by comparing the results to the control cells counted at T0.

### 2.24. Ki67 ELISA Kit

According to the manufacturer’s instructions, Ki67 activity using the ELISA kit (Abcam, San Diego, CA, USA) was determined on the co-culture prostatic model. Cold PBS1x was used for prostate lysates. After adding 50 μL of the sample and 50 μL of Antibody Cocktail to each well, the plate was incubated at room temperature for 1 h. After washing the plate with 1X Wash Buffer, 100 μL of TMB Development Solution was added to each well, and the plate was allowed to be at room temperature for 10 min. After adding 100 μL of Stop Solution, the plate was analysed at 450 nm using a spectrometer (Infinite 200 Pro MPlex, Tecan, Männedorf, Switzerland). The results were presented as means ± SD (%) against control (line 0), with the concentration represented as pg/mL against a standard curve (range 31.25–2000 pg/mL).

### 2.25. Statistical Analysis

Prism GraphPad statistical software 9.5.0 was used to process the gathered data. A one-way analysis of variance (ANOVA) and Bonferroni post hoc tests were then utilised. Student’s two-tailed *t*-test was used to compare groups. After a two-way ANOVA, a two-sided Dunnett post hoc test was used to evaluate multiple group comparisons. The results were summarised using the mean ± SD of at least five triplicate experiments. A difference was considered significant if the *p*-value fell below 0.05.

## 3. Results

### 3.1. CaCo-2 Cells’ Cell Viability in the Presence of Probiotics

**Figure 1:**
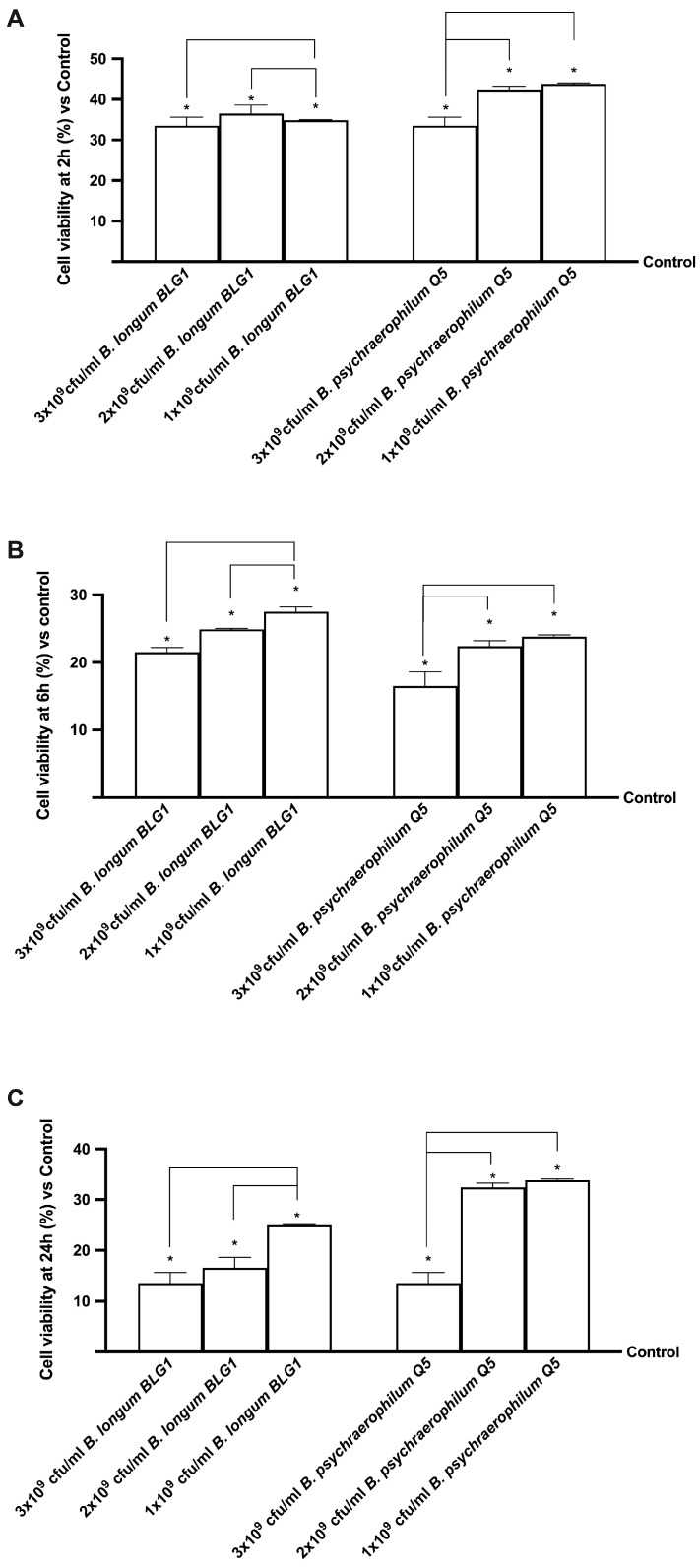
Cell viability on CaCo-2 cells cultured for 2 h (**A**), 6 h (**B**), and 24 h (**C**) with *B. longum* and *B. psychaerophilum* at varying doses. Results are based on five independent experiments conducted in triplicates, compared to control values (0% line). * *p* < 0.05 vs. control; bar *p* < 0.05 against different concentrations.

In the human intestinal cell model, *B. longum* BLG1 and *B. psychaerophilum* Q5 strains were investigated for prostatic hyperplasia reduction at various dosages. CaCo-2 cell viability was examined for 2 h, 6 h, and 24 h to estimate the human absorption time after treatment with 1 × 10^9^, 2 × 10^9^, and 3 × 10^9^ CFU/mL (10 mg, 20 mg, and 30 mg, respectively) of *B. longum* BLG1 or *B. psychaerophilum* Q5, as suggested by the Italian Health Minister’s “Guidelines on Probiotics and Prebiotics” in March 2018 [44], which recommended at least 109 live bacterial cells daily for these test dosages. Figure 1 and Table A1 shows that all the concentrations examined enhanced CaCo-2 cell viability (*p* < 0.05), excluding cytotoxic effects. *B. longum* BLG1 showed increased mitochondrial well-being at 1 × 10^9^ CFU/mL compared to the control (*p* < 0.05) and was significantly higher than 2 × 10^9^ CFU/mL and 3 × 10^9^ CFU/mL (*p* < 0.05). These results indicate that the appropriate dosage for *B. longum* BLG1 is 1 × 10^9^ CFU/mL. *B. psychaerophilum* Q5 at 1 × 10^9^ CFU/mL showed significant mitochondrial metabolism stimulation compared to the control (*p* < 0.05) and other concentrations (*p* < 0.05). Indeed, even though both 1 × 10^9^ CFU/mL and 2 × 10^9^ CFU/mL *B. psychaerophilum* Q5 produced significant results compared to 3 × 10^9^ CFU/mL *B. psychaerophilum* Q5, 1 × 10^9^ exerted an effect about 5% greater than 2 × 10^9^ at all timepoints analysed, so it was maintained for subsequent experiments. The probiotic’s capacity to promote the health of intestinal cells may be attributed to these cellular mechanisms. Therefore, in subsequent experiments, 1 × 10^9^ CFU/mL *B. longum* BLG1 and 1 × 10^9^ CFU/mL *B. psychaerophilum* Q5 were selected for further investigation.

### 3.2. In Vitro Intestinal Barrier Effects of B. longum and B. psychaerophilum

**Figure 2:**
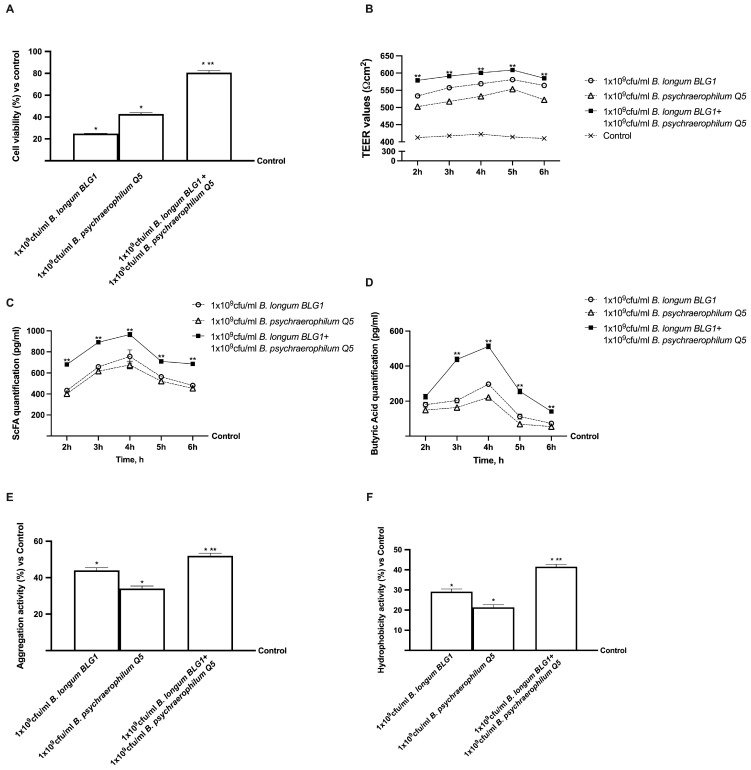
Permeability and adhesion study on CaCo-2 cells. In (**A**), Cell viability is measured by using the MTT test; in (**B**), TEER Value using EVOM3; in (**C**), SCFA analysis is conducted using an ELISA kit; in (**D**), butyric acid analysis is carried out using an ELISA kit; in (**E**) the analysis of aggregation activity and in (**F**) the analysis of hydrophobicity activity. Data are mean ± SD of five independent experiments performed in triplicates compared to control values (0% line). In (**A**,**C**–**F**), * *p* < 0.05 vs. control; ** *p* < 0.05 vs. single agents. In (**B**), *p* < 0.05 vs. control; ** *p* < 0.05 vs. single agents.

**Figure 3:**
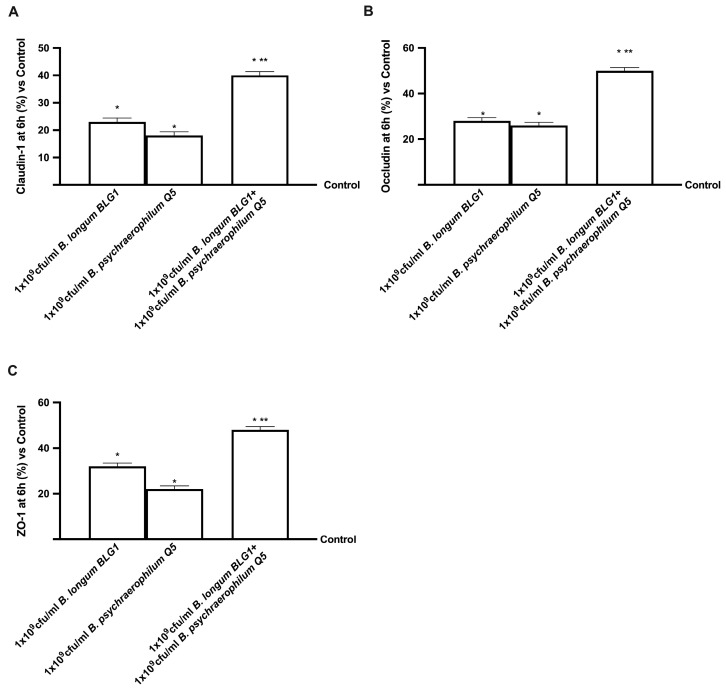
Integrity analysis. In (**A**), the analysis of claudin-1 measured by ELISA test; in (**B**), the analysis of ZO-1 by ELISA test; in (**C**), the analysis of occludin-1 by ELISA kit. Data are mean ± SD of five independent experiments performed in triplicates vs. control values (0% line) and * *p* < 0.05 vs. control; ** *p* < 0.05 vs. single agents.

In an in vitro 3D intestinal model, *B. longum* BLG1 and *B. psychaerophilum* Q5’s regulatory capacities were tested to replicate the complexity of the human intestinal barrier. TEER values, butyric acid production, cell viability, hydrophobic and aggregation activities, and combined stimulation of 1 × 10^9^ CFU/mL *B. longum* BLG1 and *B. psychaerophilum* Q5 were measured from 2 h to 6 h. According to the data presented in Figure 2A, cells treated with both probiotics showed an improvement in cell viability at 6 h when compared to both the probiotic strains evaluated alone (*p* < 0.05) and the control (*p* < 0.05). Combining 1 × 10^9^ CFU/mL *B. longum BLG1* and 1 × 10^9^ CFU/mL *B. psychraelophilum* Q5 increases cell viability by 2.2 times and 88%, respectively, compared to single agents (*p* < 0.05). The intestinal level showed the most significant benefit from the combination of 1 × 10^9^ CFU/mL *B. longum* BLG1 and 1 × 10^9^ CFU/mL *B. psychraelophilum* Q5 compared to single agents (*p* < 0.05). A comparison of TEER values (Figure 2B) revealed that probiotic strains alone had a greater basal value than the control (*p* < 0.05). The combination resulted in a significant improvement (*p* < 0.05). During all stimulation times, 1 × 10^9^ CFU/mL *B. longum* BLG1 and 1 × 10^9^ CFU/mL *B. psychraelophilum* Q5 marginally extended the positive impact compared to the other agents (*p* < 0.05), preserving permeability without changing epithelial integrity. The Claudin-1, Occludin, and Zo-1 levels were further analysed using 3D in vitro models to fully assess the intestinal barrier integrity following treatment. Additional experiments evaluated a primary SCFA generated by *B. longum* BLG1 and *B. psychaerophilum* Q5 from these promising outcomes. As illustrated in Figure 2C,D, the findings showcased the capacity of *B. longum BLG1* and *B. psychaerophilum* Q5 individually and together to generate SCFA and butyric acid compared to the control group (*p* < 0.05). Specifically, SCFA production (Figure 3A) was elevated following the combined treatment compared to the single probiotics, reaching a peak at 4 h of treatment (28% vs. 1 × 10^9^ CFU/mL *B. longum BLG1* and 43% vs. 1 × 10^9^ CFU/mL *B.psychraelophilum, p* < 0.05, *p* < 0.05). Additionally, the production of butyric acid (Figure 2D) was notably higher when 1 × 10^9^ CFU/mL *B. longum BLG1* was combined with 1 × 10^9^ CFU/mL *B. psychraelophilum* Q5 compared to the single agents, with peak production at around 24% at 4 h (*p* < 0.05). This suggests the capability of this combination to uphold proper intestinal balance and metabolite production.

Aggregation and hydrophobicity activities were examined after the stimulation period (Figure 2E,F) since they are significant variables that significantly impact bacterial adhesion. These two assays showed that the two probiotic strains have hydrophobic cell surfaces and aggregation capacity, making them better at adhering to intestinal cells. Compared to single agents, 1 × 10^9^ CFU/mL *B. longum* BLG1 and 1 × 10^9^ CFU/mL *B. psychraelophilum* Q5 had a significant effect (*p* < 0.05), indicating probiotic adherence to the gut barrier.

At the end of the stimulation time, ZO-1, claudin-1 and occluding-1 (Figure 3) confirmed the beneficial effects of all concentrations of probiotics tested and the ability to maintain the physiological function of all tight junctions selected (*p* < 0.05). As expected, 1 × 10^9^ CFU/mL *B. longum BLG1* plus 1 × 10^9^ CFU/mL *B. psychraelophilum Q5* exerted a considerable effect compared to the single agents (*p* < 0.05). Specifically, concerning Claudin-1, 1 × 10^9^ CFU/mL *B. longum BLG1* plus 1 × 10^9^ CFU/mL *B. psychraelophilum Q5* induced a more significant effect of 74% compared to ×10^9^ CFU/mL *B. longum BLG1* and 1.2 times compared to 1 × 10^9^ CFU/mL *B. psychraelophilum Q5*. For Occludin, the 1 × 10^9^ CFU/mL *B. longum BLG1* plus 1 × 10^9^ CFU/mL *B. psychraelophilum Q5* effect was about 79% higher than ×10^9^ CFU/mL *B. longum BLG1* and about 92% higher than 1 × 10^9^ CFU/mL *B. psychraelophilum Q5*. Finally, also in ZO-1 analysis, it was found that 1 ×10^9^ CFU/mL *B. longum BLG1* plus 1 × 10^9^ CFU/mL *B. psychraelophilum Q5* induced a greater effect compared to 1 × 10^9^ CFU/mL *B. longum BLG1* and 1 × 10^9^ CFU/mL *B. psychraelophilum Q5* (about 50% and 1.2 times, respectively). Thus, these data confirmed the regulatory effect of probiotics on the gut barrier.

### 3.3. Analysis of BPH In Vitro Oxidative Stress and Inflammation

**Figure 4:**
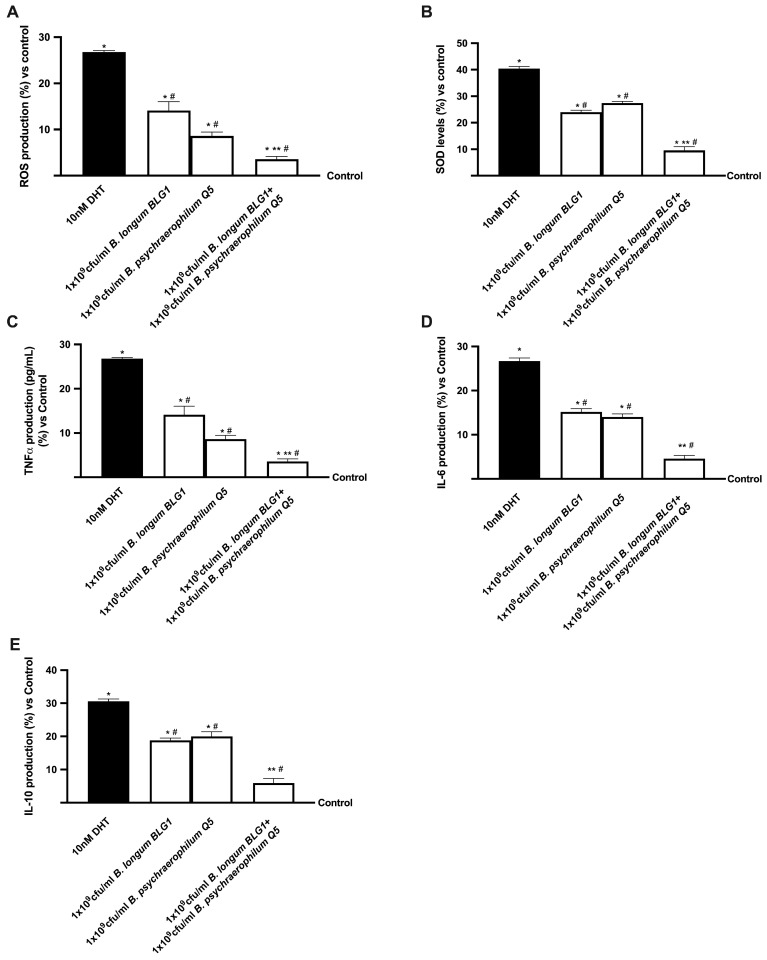
Analysis of oxidative stress and inflammation. All of the following results are derived from the ELISA kit: (**A**) ROS generation; (**B**) SOD levels; (**C**) TNF-α production; (**D**) IL-6 production; and (**E**) IL-10 production. The mean ± SD of five separate studies conducted in triplicate is shown, along with * *p* < 0.05 vs. control, ** *p* < 0.05 vs. single drugs, and # *p* < 0.05 vs. DHT, in comparison to control values (0% line).

Numerous experiments were conducted to investigate the impact of 1 × 10^9^ CFU/mL *B. longum* BLG1 and 1 × 10^9^ CFU/mL *B. psychaerophilum* Q5, both individually and in combination, on an in vitro co-culture prostatic model of BPH under hyperplasia conditions induced by the pre-treatment with 10 nM DHT for 4 days, as ROS production and interleukins activation, including TNF-α, IL6, and IL10, were critical factors in the development of BPH. By inhibiting the superoxide dismutase (SOD) antioxidant mechanism, the exposure to 10 nM DHT substantially increased ROS production (*p* < 0.05 compared to control, Figure 4A,B). Conversely, the probiotics’ capacity to mitigate the oxidative stress that is characteristic of BPH was demonstrated by the significant reduction in ROS levels and SOD activity observed following the sequential treatment with *B. longum* BLG1 and *B. psychaerophilum* Q5 alone (*p* < 0.05). Combining 1 × 10^9^ CFU/mL *B. longum* BLG1 and 1 × 10^9^ CFU/mL *B. psychraelophilum* Q5 decreased ROS production by approximately 6 times. It reduced SOD activity by approximately 3 times (*p* < 0.05) in comparison to DHT, improving these effects. Similarly, as shown in Figure 4C–E, TNF-α production is markedly elevated following DHT therapy, resembling the hyperplasia state that increases IL-6 and IL-10 secretion (*p* < 0.05 compared to control). Treatment with 1 × 10^9^ CFU/mL *B. longum* BLG1 and 1 × 10^9^ CFU/mL *B. psychaerophilum* Q5 alone restored this condition (*p* < 0.05 vs. DHT). In addition, 1 × 10^9^ CFU/mL *B. longum* BLG1 and 1 × 10^9^ CFU/mL *B. psychraelophilum* Q5 together reduce the production of pro-inflammatory cytokines (approximately 6.5 times for TNF-α, 5 times for IL-6 and IL-10, *p* < 0.05 compared to DHT), suggesting that intestinal probiotic treatment can modulate BPH damage.

### 3.4. In Vitro Hyperplasia Mitigation by B. longum and B. psychaerophilum

**Figure 5:**
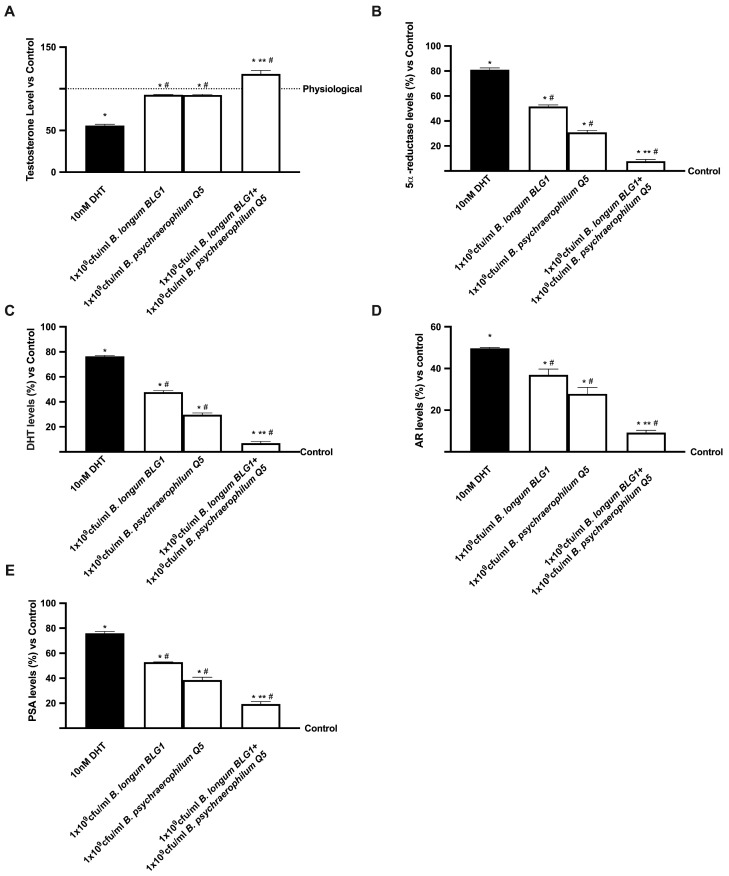
Mechanism analyses for prostate hyperplasia. Testosterone levels (**A**), 5α-reductase levels (**B**), DHT levels (**C**), AR levels (**D**), and PSA levels (**E**) are all derived from the ELISA kit. Values are mean ± SD from five independent tests in triplicates compared to control values (0% line). * *p* < 0.05 vs. control; ** *p* < 0.05 vs. single agents; # *p* < 0.05 vs. DHT.

Additionally, a co-culture prostatic model examined the key intracellular pathways involved in BPH to determine how 1 × 10^9^ CFU/mL *B. longum* BLG1 and 1 × 10^9^ CFU/mL *B. psychaerophilum* Q5 can alleviate prostate hyperplasic conditions. 5α-reductase 2 converts 10 nM DHT to DHT once it reaches prostatic tissue, as seen in Figure 5A–D. Consequently, DHT increases AR on prostate cells (*p* < 0.05 vs. control), causing uncontrolled changes in prostatic processes. However, when compared to 10 nM DHT, the single administration of 1 × 10^9^ CFU/mL *B. longum* BLG1 and 1 × 10^9^ CFU/mL *B. psychaerophilum* Q5 significantly decreased 5α-reductase two levels (about 36% for 1 × 10^9^ CFU/mL *B. longum* BLG1 and 56% for 1 × 10^9^ CFU/mL *B. psychaerophilum* Q5, *p* < 0.05). Consequently, DHT production decreases significantly, reducing AR activity (*p* < 0.05 vs. 10 nM DHT). Combining 1 × 10^9^ CFU/mL *B. longum* BLG1 and 1 × 10^9^ CFU/mL *B. psychraelophilum* Q5 with 10 nM DHT improved benefits (*p* < 0.05), primarily due to a single probiotic strain. The combination’s beneficial effects were amplified by inhibiting 5α-reductase levels (9 times, *p* < 0.05), decreasing DHT production (10 times, *p* < 0.05), and reducing AR levels (4 times, *p* < 0.05) compared to pre-treatment. These findings suggest that probiotics could modulate BPH’s molecular process, which causes hyperplasia. Research shows that the presence of DHT in the AR region helps cells grow, as shown by the amount of PSA in the blood. Therefore, to determine if probiotics can reduce PSA production during BPH, an ELISA reagent was used to test 1 × 10^9^ CFU/mL *B. longum* BLG1 and 1 × 10^9^ CFU/mL *B. psychaerophilum* Q5 alone and in combination. As expected, 10 nM DHT increased PSA by 76% compared to the control (*p* < 0.05). In the presence of 1 × 10^9^ CFU/mL *B. longum* BLG1 and 1 × 10^9^ CFU/mL *B. psychaerophilum* Q5, PSA levels were significantly lowered by 31% and 50%, respectively, compared to BPH (*p* < 0.05). Combining 1 × 10^9^ CFU/mL *B. longum* BLG1 and 1 × 10^9^ CFU/mL *B. psychraelophilum* Q5 showed a significant 75% improvement (*p* < 0.05) over 10 nM DHT.

### 3.5. B. longum and B. psychaerophilum’s Capacity to Impede the Progression of BPH

**Figure 6:**
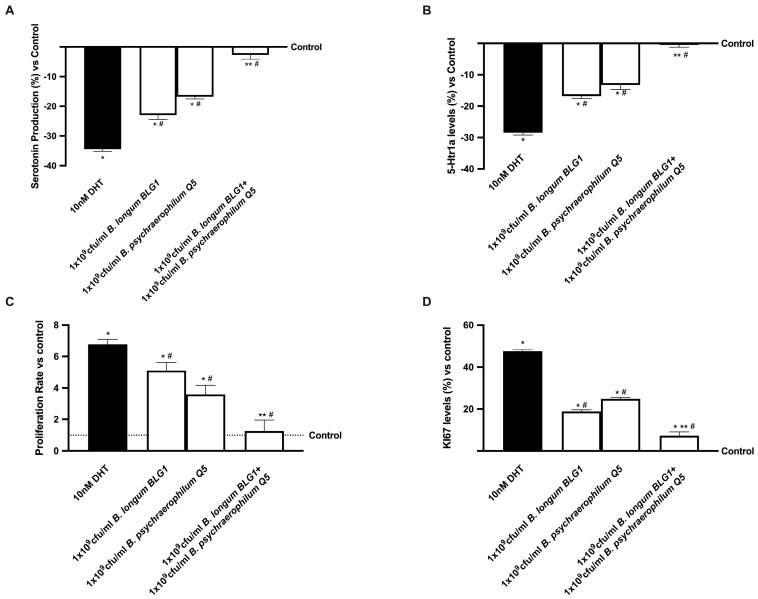
Serotonin and proliferation studies. The following results are derived from an ELISA kit: (**A**) 5-Htr1a levels, (**B**) serotonin production, (**C**) Ki67 expression, and (**D**) proliferation rate. The mean ± SD of five separate, duplicate tests is presented, along with * *p* < 0.05 vs. control, ** *p* < 0.05 vs. single drugs, and # *p* < 0.05 vs. DHT, in comparison to control values (0% line).

According to current research, AR is activated when serotonin is depleted in prostatic cells. In the BPH model, *B. longum* BLG1 and *B. psychaerophilum* Q5 were tried separately and in combination to restore serotonin synthesis. In addition, 10 nM DHT significantly (*p* < 0.05) reduced serotonin production, according to the BPH model (Figure 6A), but 1 × 10^9^ CFU/mL *B. longum* BLG1 and 1 × 10^9^ CFU/mL *B. psychaerophilum* Q5 by themselves almost brought serotonin production back to control levels (*p* < 0.05). Treatment with 1 × 10^9^ CFU/mL *B. longum* BLG1 and 1 × 10^9^ CFU/mL *B. psychraelophilum* Q5 resulted in higher serotonin levels than single strains (88% and 87%, respectively, *p* < 0.05). Serotonin’s inhibitory growth function through 5-Htr1a to reduce prostatic hyperplasia was confirmed. As shown in Figure 6B, 10 nM DHT-induced hyperplasia resulted in lower serotonin receptor levels (*p* < 0.05 vs. control), while 1 × 10^9^ CFU/mL *B. longum* BLG1 and 1 × 10^9^ CFU/mL *B. psychaerophilum* Q5 significantly increased receptor activity (*p* < 0.05 vs. 10 nM DHT). Combining 1 × 10^9^ CFU/mL *B. longum* BLG1 and 1 × 10^9^ CFU/mL *B. psychraelophilum* Q5 significantly amplifies the effect. During BPH, this combination may maintain intestinal homeostasis and inhibit AR upregulation.

Additionally, cell growth was examined by analysing the proliferation rate and Ki67 expression in the same condition. The study found that 1 × 10^9^ CFU/mL *B. longum* BLG1 and 1 × 10^9^ CFU/mL *B. psychaerophilum* Q5 significantly reduced Ki67 expression (Figure 6D) compared to 10 nM DHT (*p* < 0.05), indicating cell growth inhibition, as shown by crystal violet staining (Figure 6C). The combination of 1 × 10^9^ CFU/mL *B. longum* BLG1 and 1 × 10^9^ CFU/mL *B. psychraelophilum* Q5 (*p* < 0.05) effectively inhibited Ki67 expression and cell proliferation, proving that the formulation outperforms single agents (*p* < 0.05).

## 4. Discussion

The high prevalence of BPH has become a significant global health issue that impacts elderly male populations [45]. Nonetheless, there is no effective treatment for BPH. 5 α-reductase inhibitors, including finasteride and dutasteride, are the most frequently prescribed medications. Nevertheless, the primary cause of BPH is not resolved by the use of α-blockers, as they only relax the smooth muscle of the prostatic urethra and do not alleviate prostatic pathogenesis, negatively impacting the quality of life [46]. Numerous illnesses are believed to connect with the gut microbiota, which is seen as a dynamic system impacting human health [47]. Indeed, various research has indicated that the gut microbiome is strongly linked to numerous diseases outside the intestines, such as those affecting the urinary tract [48,49]. The impact of the gut microbiome on prostate ailments is widely acknowledged, supported by increasing evidence of a gut–prostate connection [13,50]. This proof highlights the intricate connection between the gut and other organs, stressing the significance of the gut–organ axis and inter-organ communication. Despite the considerable distance between the gut and prostate and the lack of a known link between gut microbiota and the prostate until recently, new research revealed that the ratio of Firmicutes to Bacteroidetes in the human gut microbiota is associated with prostate enlargement. When the immune system is activated and pro-inflammatory cytokines (IL-17, IL-23, TNF-α, IFN-γ) are released, they can affect different processes in the host organism. This shows the significance of the gut–prostate axis [12]. These cytokines may play a role in developing inflammatory conditions [51]. It has been demonstrated that there are notable differences in GM’s functional profile and content between healthy and sick people. The presence of SCFA-producing bacteria, such as Bifidobacterium species, is, for instance, underrepresented in people with cardiometabolic and autoimmune disorders. On the other hand, individuals who suffer from atopic eczema or recurrent urinary tract symptoms (rUTS) frequently harbour harmful microorganisms, such as Escherichia coli, Staphylococcus aureus, and Clostridium difficile [51]. Additionally, Romano et al. have demonstrated that a substantial number of celiac disease patients experience sexual dysfunction, which appears to be associated with intestinal inflammation and urinary tract dysfunction [52]. Consequently, the “gut–distant organ axis” theory describes the approach of connecting the gut with distant organs, which posits that a pro-inflammatory state is the primary connection between prostate disease and the permeability of the intestinal epithelial barrier (IEB) [13,50]. The concept of the gut–prostate axis, which describes the intimate connection between the gut and the prostate in treating prostatitis, was initially proposed in 2005 [15]. Numerous investigations have since shown a link between abnormalities of GM amount and function and prostate disease. According to clinical studies and ongoing research, intestinal inflammation and functional status are risk factors for prostate pathology development. GM may be a complementary treatment for both disease groups in these cases [53,54].

Despite being recognized as a complex inflammatory disorder that causes both morphological and functional alterations, no single factor has been found to contribute to the chronic inflammatory response in BPH directly. On the other hand, intestinal microbiota dysfunctions impact prostatic inflammation [55]. Critical regulators of metabolic inflammation and alteration have been identified in the gastrointestinal tract and its bacterial inhabitants [56]. Numerous reviews have already reported correlations between oral microbial dysfunction and various diseases; however, the mechanistic role of intestinal microbiota in disease progression has not yet been clarified [21]. There is substantial evidence from many studies that an intestinal inflammatory microenvironment is responsible for developing tissue lesion precursors that facilitate the initiation of BPH [57]. Even though gastrointestinal resident microorganisms may not directly damage the prostate, the imbalance of bacterial metabolite production may impact the health of the intestinal tract and other organs, including the prostate. SCFAs are an example of bacterial metabolites that have been demonstrated to significantly promote human homeostasis by acting as mediators between the intestinal microbiota and host cells in this context. Therefore, quantifying SCFAs in the stool may yield insights into the gastrointestinal microbiota and its potential interactions with other tissues and organs [10]. As a result, the current investigation developed an in vitro model to investigate the impact of probiotics supplementation on prostatic hyperplasia for the first time. This investigation demonstrates that it is conceivable to enhance the reduction in BPH by regulating the primary intracellular pathway and influencing the equilibrium of metabolites in the intestinal microbiota. A prostatic model was used to recreate the pathophysiology of BPH better and accomplish this goal. In this BPH model, probiotics with anti-inflammatory qualities were used to evaluate intrinsic homeostasis and alter the prostate environment to reduce the disease. The findings indicate that the inflammatory response characteristic of BPH can be mitigated by the combination of 1 × 10^9^ CFU/mL *B. longum* BLG1 and 1 × 10^9^ CFU/mL *B. psychraerophilum* Q5, which occurs by modulating 5 α-reductase activity and reducing DHT production. It is important to mention that Sarkar et al. [57] recently demonstrated a difference in the composition of the intestinal microbiome in men undergoing BPH treatment. Furthermore, this investigation demonstrated a significant improvement in the properties of the gut microbiota, particularly the combined probiotics’ ability to promote intestinal health, thereby emphasising a synergistic effect. The mixtures were more effective at protecting the integrity of TJ dynamics in intestinal cells, which proved that these compounds are safe. Additionally, SCFA production was assessed due to its immunomodulatory potential and ability to maintain an anti-inflammatory equilibrium. They modulate the immune response by influencing intestinal immune cells and acting locally in the intestine colonised by commensal bacteria. This is accomplished by creating multiprotein inflammasome complexes found all over the body [19]. As anticipated, the treatment with 1 × 10^9^ CFU/mL *B. longum* BLG1 and 1 × 10^9^ CFU/mL *B. psychraerophilum* Q5 results in SCFA variations, specifically butyric acid, which serve as evidence of their capacity to preserve intestinal homeostasis. Furthermore, the experiments emphasised the bacterial strains’ adhesion capacity, which confirmed that the probiotics affected their activity through metabolite production without crossing the intestinal barrier. Moreover, intriguing evidence was discovered during the examination of the effect of 1 × 10^9^ CFU/mL *B. longum* BLG1 and 1 × 10^9^ CFU/mL *B. psychraerophilum* Q5, both individually and in combination after oral ingestion, on a co-culture prostatic model under BPH conditions. The findings of our investigation emphasised the significance of DHT treatment in the development of hyperproliferation and hyperplasia, as it increases the expression of numerous parameters, such as inflammatory mediators, inflammatory genes, and oxidative stress. In addition, prostatic inflammation may induce the activation of the nuclear transcription factor NF-κB through the TNF-α transduction pathway and the generation of free radicals, including ROS [58]. Conversely, the antioxidant activity was enhanced and oxidative stress was reduced by the oral supplementation of 1 × 10^9^ CFU/mL *B. longum* BLG1 and 1 × 10^9^ CFU/mL *B. psychraerophilum* Q5, individually and in combination. Moreover, the novel formulation reduced TNF-α and IL-6/IL-10 levels, showing its ability to reduce BPH-induced inflammation and damage. Specifically, prior research has demonstrated that the prostate tissues of experimental rodents with age-induced BPH exhibit elevated expression levels of anti-inflammatory cytokines, such as IL-10. As a result, a decrease in the expression of IL-10 in the BPH-induced condition may indicate the possibility of reducing BPH-induced prostate inflammation [59].

Additionally, it was shown that 1 × 10^9^ CFU/mL *B. longum* BLG1 + 1 × 10^9^ CFU/mL *B. psychraerophilum* Q5 can regulate the conversion of testosterone into DHT by 5α-reductase. Since they were able to bring 5α-reductase and DHT levels back to levels close to physiological levels, SCFAs produced by 1 × 10^9^ CFU/mL *B. longum* BLG1 plus 1 × 10^9^ CFU/mL *B. psychraerophilum* Q5 could potentially be used as an alternative for treating this condition. This would help counteract the hyperactivity brought on by the DHT-mimicking hyperplasia condition. Furthermore, the probiotics currently being studied are of even greater significance due to their capacity to modulate AR activity, which underscores their efficacy in most critical prostate function processes and in pathological conditions like BPH. This hypothesis is further supported by the evidence of their capacity to reduce PSA levels, a result typically obtained following surgical treatment of BPH. Specifically, 1 × 10^9^ CFU/mL *B. longum* BLG1 and 1 × 10^9^ CFU/mL *B. psychraerophilum* Q5 have been shown to achieve this target. The activity of AR is also associated with serotonin, a potent negative regulator of prostate growth. AR downregulation is linked to this adverse regulatory impact [60]. The aetiology of BPH has been associated with serotonin depletion, which is mediated by the modulation of AR. Compared to normal prostate tissue, BPH tissue either lacks serotonin or has less of the neuroendocrine cells that secrete it [60]. In this context, 1 × 10^9^ CFU/mL *B. longum* BLG1 and 1 × 10^9^ CFU/mL *B. psychraerophilum* Q5 demonstrated the capacity to regulate serotonin production when used individually or in combination. Although this is not a synergistic effect, it is plausible. In conclusion, the capacity of the combination of selected probiotic strains to contain altered growth, which is one of the primary issues associated with BPH, illustrates their actual potential for use in BPH management.

## 5. Conclusions

For the first time, the study demonstrated that the reduction in BPH can be facilitated by affecting the equilibrium of metabolites in the intestinal microbiota. Additional extensive research is necessary to elucidate further the potential mechanisms and causal relationships between BPH and gut microbiota and provide a more detailed mechanism by which intestinal commensal bacteria affect BPH. The study’s use of an in vitro model, already well-established and utilised in the literature for these studies, is a specific limitation. Despite this, the model must be able to replicate the disease’s complexity accurately. Consequently, additional in vivo investigations will be required. It may be suggested that the microbiota and/or its metabolites serve as new indicators and potential treatment targets for BPH. Nevertheless, the mechanisms by which the healthy intestinal microbiota, in conjunction with probiotic supplementation, mitigate hyperplasia progression may provide an alternative approach to further investigating the microbiome signature as a coadjutant therapeutic strategy against BPH.

## Data Availability

The data presented in this study are available on request from the corresponding author (The Laboratory of Physiology carefully stores raw data to ensure permanent retention under a secure system).

## Abbreviations

5-HT	Serotonin
5-Htr1a	5-hydroxytryptamine 1a
ANOVA	One-way analysis of variance
AR	Androgen receptor
*B. longum*	*Bifidobacterium longum* subsp. *longum* novaBLG1 DSM 34338
*B. psychraerophilum*	*Bifidobacterium psychraerophilum* Q5 DSM 33131
BPH	Benign prostatic hyperplasia
CaCo-2	Human immortalized colorectal adenocarcinoma cell line
DHT	Dihydrotestosterone
DMEM	Dulbecco’s Modified Eagle’s Medium
DMEM-F12	Dulbecco’s Modified Eagle’s Medium/Nutrient F-12 Ham
DSM	German Collection of Microorganisms and Cell Cultures
EGF	Epidermal growth factor
ELISA	Enzyme-Linked Immunosorbent Assay
EMA	European Medicines Agency
FBS	Foetal bovine serum
FDA	Food Drug Administration
GM	Gut microbiome
HBSS	Hanks’s Balanced Salt solution
IEB	Intestinal epithelial barrier
IFN- γ	Interferon- gamma
IL-10	Interleukin 10
IL-17	Interleukin-17
IL-23	Interleukin-23
IL-6	Interleukin-6
IL-8	Interleukin-8
MTT	3-(4,5-Dimethylthiazol-2-yl)-2,5-Diphenyltetrazolium Bromide
PBS	Phosphate-buffered saline
PSA	Prostate-Specific Antigene
ROS	Reactive oxygen species
rUTS	Recurrent urinary tract symptoms
RWPE-1	Normal prostate epithelial cell
SCFA	Short-chain fatty acid
SOD	Superoxide dismutases
SRD5A2	3-oxo-5-alpha-steroid 4-dehydrogenase
TEER	Transepithelial Electrical Resistance
TNF-α	Tumor Necrosis Factor-alpha
UPO	University of Eastern Piedmont
WPMY-1	Normal prostate stroma fibroblast cell

## Appendix A

**Table A1:** Time course dose–response study of cell viability using MTT assay on CaCo-2 cells.

	3 × 10^9^ cfu/mL *B. longum BLG1*	2 × 10^9^ cfu/mL *B. longum BLG1*	1 × 10^9^ cfu/mL *B. longum BLG1*	3 × 10^9^ cfu/mL *B. psychraerophilum Q5*	2 × 10^9^ cfu/mL *B. psychraerophilum Q5*	1 × 10^9^ cfu/mL *B. psychraerophilum Q5*
2 h	33.4% ± 2.1	36.6% ± 2.0	33.4% ± 2.1	33.6% ± 2.1	42.4% ± 0.8	43.8% ± 0.5
6 h	21.5% ± 0.8	24.9% ± 0.1	27.6% ± 0.7	16.4% ± 2.0	22.4% ± 0.8	23.9% ± 0.3
24 h	13.6% ± 2.0	16.4% ± 2.1	24.9% ± 0.1	13.6% ± 1.8	32.5% ± 0.9	33.8% ± 0.4
